# Treatment of Fistula in Ano

**Published:** 1911-01-21

**Authors:** A. B. Fearnley


### The General Practitioner's Column.

[Contributions to this Column are invited, and if accepted, will be paid for.]

### TREATMENT OF FISTULA IN ANO.

By A. B. FEARNLEY, M.B., B.S. Lond.

The operation for fistula is a very simple one, and may be readily undertaken by the general practitioner; and yet, unless properly performed with thorough attention to detail, it will not only certainly fail but will leave the patient in a worse condition than before.

The first point for consideration is the nature of the fistula. Is it complete, blind external, or blind internal? When once the probe has established the fact of the sinus, the diagnosis of the variety of fistula may be left till the operation, since an operation will in any case be necessary.

The method of procedure should be as follows: The bowels should be thoroughly emptied by means of aperients for three or four nights, if possible, before the operation, and an enema on the night preceding and again on the morning of the operation some three or four hours before the time decided on. The patient having been anæsthetised and placed in the lithotomy position, the part is shaved and subjected to the usual pre-operative ritual of cleansing.

A probe is then passed into the sinus and a finger introduced into the rectum—it is important to pass the probe first, and then the finger, since the passage of the latter causes a contraction of the external sphincter and obliteration of the lumen of the sinus. The finger is now used as a guide, and a search made for the internal opening—remembering that the fistulous tract is probably branched and that the internal opening may be situated at the termination of one of the branches. It is of extreme importance to be certain of the non-existence of the internal opening before deciding to treat the case as one of blind external fistula, since if the internal opening is missed the whole operation will be in vain. If the probe be felt presenting under the mucous membrane of the rectum, it should be pushed through, converting the incomplete into the complete variety.

Supposing an internal opening is discovered or made—then, with the probe still in position, a director is passed along the track into the rectum, and the probe is withdrawn. One blade of a pair of scissors is now passed in the groove of the director, and the point being guarded by the finger in the rectum, the tissues between the director and the skin are cut clean through, usually dividing the external sphincter in the process.

The most important step in the operation now commences, and the part which is most frequently overlooked—namely, the search for branches of the fistulous track. Whenever any such is found, the director is passed along it and the tissues divided with the scissors exactly as has been already described—it is impossible to lay too much stress on the importance of this step. Finally, all the fistulous tracts are very thoroughly scraped with a sharp spoon, and packed to the base with iodoform ribbon gauze. If no internal opening can be found the director is passed and the tissues divided with the scissors as before, the same care being taken to discover and lay open any branches—the unhealthy tissue is scraped away as before, and the whole is plugged and secured with gauze and T bandage.

In the case of blind internal fistula the opening is discovered by the finger in the rectum, a curved probe is passed into it and as far as possible towards the skin, when it is cut down on with a scalpel, thus converting the condition into complete fistula, when the further treatment will be as has been already described.

After the operation the bowels should be kept confined for three or four days, especially where the complete operation has been performed. The wound should be dressed twice daily, and the greatest care taken to plug down to the bottom of the wound in order to ensure sound healing from the base.

